# Details acquired from medical history and patients’ experience of empathy – two sides of the same coin

**DOI:** 10.1186/1472-6920-13-67

**Published:** 2013-05-09

**Authors:** Friedemann Ohm, Daniela Vogel, Susanne Sehner, Marjo Wijnen-Meijer, Sigrid Harendza

**Affiliations:** 1 University Medical Centre Hamburg-Eppendorf, Department of Internal Medicine, Martinistr. 52, 20246 Hamburg, Germany; 2 University Medical Centre Hamburg-Eppendorf, Institute for Biometrics and Epidemiology, Martinistr. 52, 20246 Hamburg, Germany; 3 Center for Research and Development of Education, UMC Utrecht, P.O. Box 85500, Utrecht, GA 3508 The Netherlands

**Keywords:** History taking, Medical history, Communication, Competence, Empathy, Feedback

## Abstract

**Background:**

History taking and empathetic communication are two important aspects in successful physician-patient interaction. Gathering important information from the patient’s medical history is needed for effective clinical decision making while empathy is relevant for patient satisfaction. We wanted to investigate whether medical students near graduation are able to combine both skills as required in daily medical practice.

**Methods:**

Thirty near graduates from Hamburg Medical School participated in an assessment for clinical competences including a consultation hour with five standardized patients. Each patient interview was videotaped and standardized patients rated participants with the CARE questionnaire for consultation and relational empathy. All videotaped interviews were rated with a checklist based on the number of important medical aspects for each case. Data were analysed with the linear mixed model to correct for random effects. Regression analysis was performed to look for correlations between the number of questions asked by a participant and their respective empathy rating.

**Results:**

Of the 123 aspects that could have been gathered in total, students only requested 56.4% (95% CI 53.5-59.3). While no difference between male and female participants was found, a significant difference (p < .001) was observed between the two parts of the checklist with 61.1% (95% CI 57.9-64.3) of aspects asked for in part 1 (patient’s symptoms) versus 52.0 (95 47.4-56.7) in part 2 (further history). All female standardized patients combined rated female participants (mean score 14.2, 95% CI 12.3-16.3) to be significantly (p < .01) more empathetic than male participants (mean score 19.2, 95% CI 16.3-22.6). Regression analysis revealed no correlation between the number of medical aspects gathered by a participant and his or her respective empathy score given by the standardized patient in the CARE questionnaire.

**Conclusion:**

Gathering sufficient medical data from a patient’s history and empathetic communication are two completely separate sides of the coin of history taking. While both skills have to be acquired during medical school training with particular focus on their respective learning objectives, medical students need to be provided with additional learning and feedback opportunities where they can be observed exercising both skills combined as required in physicians’ daily practice.

## Background

Skilled history taking is still regarded to be of fundamental importance for clinical decision making [1]. According to the Nobel Peace Price laureate Bernard Lown medical history provides sufficient information in about 75% of patient encounters to make the diagnosis before performing a physical examination and additional tests [2]. One study revealed that a diagnosis which agreed with the final one was made after reading the referral letter and taking the history in 66 out of 80 new patients (i.e. 82.5%) [3]. Another study showed that history alone led to the final diagnosis in 76% of patients [4]. While an extensive amount of data on history taking skills was gathered between the 1970s and 1990s [5,6] the connection between the acquired history-content and communication skills became a centrepiece of educational research [7].

Physicians’ biomedical knowledge and understanding of the pathophysiology of diseases seem to permit appropriate hypotheses which subsequently prompt further questions to explore a patient’s history thoroughly [8]. However, patient satisfaction has been found to be linked to structural aspects of patient centred communication such as signposting, summarization and repetition [9] and also to physicians’ friendliness and empathy [10]. Underscored by a study where first-year students admitted to have struggled to communicate professionally with patients because they felt a lack of clinical knowledge and the skill to express empathy, the combination of both qualities is required in physicians’ daily practice [11]. A study from 1970 showed that fourth-year students obtained more factual information from patients than first-year students [12]. With regard to communication skills, more senior students received higher patient-satisfaction ratings compared with second-year students but no details on the amount of gathered factual information was provided in this study [13]. However, patients wish for an effective dialog with their physician, involvement in treatment decisions, and authentic caring in clinical relationships [14-16].

Even though medical history taking is a skill that physicians refine during clinical practice, the graduating student is presumed to be reasonably proficient in history taking to elicit important medical details for the clinical reasoning process and to be able to express appropriate empathy in the patient-physician communication. The traditional and not very valid method to assess the quality of history taking is - if at all - an oral presentation or a review of the written history [8]. Even more modern assessment techniques like OSCE [17] or interviews with standardized patients [18] may not be testing the quality of the gathered medical information for further clinical reasoning and the expressed empathy because of their procedural focus on communication skills. Whether a correlation exists between the factual details acquired by a physician during history taking and the empathy of the physician that patients feel during the consultation is also not known. Therefore, our aim was to study the number of clinical details medical students near graduation collected during a simulated consultation hours with standardized patients and to find out whether it is associated with the empathy perceived by patients. The research question of our study was: Does the amount of medical details gathered by medical students near graduation during history taking correlate with the students’ empathy felt by the standardized patients during the consultation?

## Methods

In the traditional six year undergraduate medical curriculum (two pre-clinical years, three clinical years organized in six thematic blocks and one final practice year) at Hamburg Medical School [19] history taking skills are taught in seminars in year two. These skills are practised in the years three to six in bedside teaching courses with real patients. Furthermore, empathetic communication is taught and supported with practical exercises in courses with standardized patients (e.g. breaking bad news) in the years three to five.

In July 2011, 30 medical students (22 female, eight male) recruited on a “first-come, first-served” basis from a cohort of 147 medical students near graduation from the medical faculty of Hamburg University participated in an assessment of clinical competences developed for the comparison of students from different types of medical curricula [20]. This assessment included a consultation hour with five standardized patients (three female, two male) as described in Table 1. Each consultation lasted for ten minutes and was videotaped. After each consultation every standardized patient assessed the student’s empathy with the German version of the “Consultation and Relational Empathy” (CARE) questionnaire [21]. Each item of the CARE questionnaire was rated on a 5-point Likert scale (1: “I totally agree” to 5: “I totally disagree”). A list of all items of the CARE questionnaire is provided in Table 2. Items 9 and 10 were excluded from calculations for the CARE questionnaire because they focus on making action plans together with the patients which was not part of the initial step of this assessment. A second phase (3 hours) for information gathering, lab requests and other test to determine differential diagnoses and to draw up a management plan for every patient and a presentation of each patient to the supervisor followed the consultation hour and was assessed separately [20]. Cronbach’s α for the CARE questionnaires with eight items was α = .90. The standardized patients, three for each role, received extensive training for their respective role and for filling out the CARE questionnaire using ratings of video taped history taking [20]. They did not know the participating students from previous encounters.

**Table 1:** Patient cases

	**Case description**	**Diagnosis**
**Case 1**	5-year-old girl with fatigue and abdominal pain (case was presented by her worried mother)	Coeliac disease
**Case 2**	53-year-old man with increasing weakness and haemoptoe	Wegener’s granulomatosis
**Case 3**	58-year-old woman with abdominal pain	Perforated sigmoid diverticulitis
**Case 4**	65-year-old woman with difficulties to speak and to swallow (accompanied by her husband)	Myasthenia gravis
**Case 5**	36-year-old man with rheumatoid arthritis and fever	Varicella zoster infection

Description and diagnoses of the five patient cases.

**Table 2:** Items of the CARE questionnaire

**Item number**	**Description**
1	Did the doctor make you feel at ease?
2	Did the doctor let you tell your story?
3	Did the doctor really listen to you?
4	Was the doctor interested in you as a whole person?
5	Did the doctor fully understand your concerns?
6	Did the doctor show care and compassion?
7	Was the doctor positive and encouraging?
8	Did the doctor explain things clearly?
9	Did the doctor help you to find a way to cope with your disease?
10	Did the doctor make a plan of action with you?

Items of the CARE questionnaire translated according to Neumann et al. [21].

Based on the role descriptions of the five patient scenarios a checklist of medical details which the students should have ascertained for further hypothesis generation was developed for each case. According to a study by Nendaz et al. [22] every checklist was divided in part 1 (patient’s symptoms) and part 2 (further history). For every item the assessor of the videotaped conversations had three options to tick off: 1) the student asked for a particular piece of information (e.g. whether the patient had a history of smoking), 2) the student did not ask for this information, 3) the standardized patient provided a particular piece of information without having been asked. When a standardized patient provided a particular piece of information and the student asked an additional question regarding this piece of information the first option was ticked off as well. Internal validity of the checklists was scrutinized in a pilot by FO and SH assessing a sample of 10 and 15 videos, respectively, showing an agreement of 91% for the second round. The 150 videos were subsequently assessed by FO with the respective checklists. At the time of assessment FO was blinded to the results of the CARE questionnaires.

Statistical analyses were performed using IBM SPSS Statistics 19.0 (SPSS Inc, Chicago, USA). To estimate the relationship between the number of questions asked by a participant (checklists) and his or her empathy rating (CARE score) a random intercept model was fitted. For adjustment of the cluster structure, resulting from the multiple measurement (5 cases per student with ratings from case 4 being calculated separately from the standardized patient, 4.1, and her husband, 4.2) students were included as a random effect. Further variables of interest which were modelled as fixed effects were students’ gender and standardized patients’ gender. Additionally, an interaction term of these variables was included in the model. To fulfil the assumption of the model, the CARE score needed to be logarithmized. Adjusted means and 95%-confidence intervals (CI) are reported. The level of significance was set to α = 0.05. Outcome measures were defined during the time of the design of the assessment. We wished to explore whether there was a difference between male and female students in overall empathy and number of medical items elucidated in history taking. Furthermore, we wished to define whether the history taking content differed for the individual cases and whether there is a correlation between the number of items requested and the empathy felt by the standardized patient. This study was performed in accordance with the Declaration of Helsinki and approved by the Ethics committee of the Medical Association of the city of Hamburg, Germany, reference number PV3649.

## Results

On average, participants asked for 56.4% (95% CI 53.5-59.3) of the 123 important medical aspects they could have gathered as important details for clinical reasoning on the standardized patients’ histories (Table 3). In part 1 (patient’s symptoms, n = 60) 61.1% of medical aspects were gathered and 52.0% of aspects in part 2 (further history, n = 63). There was no significant difference between female and male participants. In case 3 (58-year-old woman with abdominal pain), participants asked significantly more questions (70.9%) than in any other case (p < .01). Such a difference also existed for part 1 of scenario 3 (78.6%) compared to all other scenarios (p < .05). In total, significantly more questions were asked from part 1 than from part 2 (p < .05).

**Table 3:** Percentage of history questions asked by participants

	**Total**		**Part 1**		**Part 2**	
	**Mean (%)**	**95% CI**	**Mean (%)**	**95% CI**	**Mean (%)**	**95% CI**
**Total**	56.4	53.5 – 59.3	**61.1**°	57.9 – 64.3	52.0	47.4 – 56.7
**Female**	58.3	55.3 – 61.3	61.8	58.5 – 65.1	55.3	50.6 – 60.1
**Male**	54.5	49.5 – 59.6	60.4	55.1 – 65.7	48.8	41.3 – 56.2
**Case 1**	56.9	52.8 – 61.0	59.9	53.5 – 66.3	54.0	45.9 – 62.1
**Case 2**	56.4	52.3 – 60.5	64.8	58.4 – 71.1	48.9	40.8 – 57.0
**Case 3**	70.9*	66.9 – 74.9	78.6**	72.7 – 84.5	64.3***	56.9 – 71.7
**Case 4**	46.6	42.5 – 50.8	50.1	43.7 – 56.5	42.7	34.7 – 50.8
**Case 5**	51.3	47.3 – 55.3	52.1	45.6 – 58.7	50.3	42.1 – 58.5

Part 1 = patient’s symptoms, Part 2 = further history. *: Significant difference versus all cases (p < .01), **: Significant difference versus all cases (p < .05), ***: Significant difference versus case 4 (p < .05). °: Significant difference part 1 versus part 2 (p < .05).

For all cases, standardized patients voluntarily presented 17.2% (95% CI 14.9-19.5) of the 123 aspects of their histories without having been asked. Significantly more aspects from part 1 were presented to male versus female participants (29.8%, 95% CI 25.3-34.4, versus 25.9%, CI 95% 22.0-29.8, p < .05) (Table 4). The patient from case 3 reported significantly fewer aspects voluntarily compared with case 4 and 5 (p < .01) and the patient from case 1 reported significantly fewer aspects than the patient in case 4 (p < .05). While no significant differences of voluntarily reported aspects could be found for part 2, differences of similar significances as described above for the total number of aspects could be found for part 1. Overall, standardized patients volunteered significantly more information from part 1 compared with part 2 (p < .01). The total amount of aspects which standardized patients presented voluntarily without subsequently being asked about again was 8.4%.

**Table 4:** Percentage of aspects voluntarily presented by standardized patients

	**Total**		**Part 1**		**Part 2**	
	**Mean (%)**	**95% CI**	**Mean (%)**	**95% CI**	**Mean (%)**	**95% CI**
**Total**	17.2	14.9 – 19.5	**27.9°**	24.0 – 31.7	6.5	4.6 – 8.4
**Female**	16.5	14.2 – 18.9	25.9	22.0 – 29.8	6.8	4.9 – 8.7
**Male**	17.9	15.2 – 20.6	**29.8**^**#**^	25.3 – 34.4	6.1	3.8 – 8.4
**Case 1**	12.1**	6.9 – 17.4	20.0**	11.3 – 28.7	5.5	1.2 – 9.7
**Case 2**	16.6	11.3 – 21.8	25.5	16.8 – 34.1	9.1	4.8 – 13.3
**Case 3**	6.9*	2.3 – 11.6	13.8*	6.1 – 25.5	1.7	-2.1 – 5.5
**Case 4**	26.9	21.6 – 32.1	42.1	33.4 – 50.8	7.7	3.5 – 12.0
**Case 5**	23.5	18.2 – 28.8	37.9	29.1 – 46.6	8.4	4.1 – 12.7

Part 1 = patient’s symptoms, Part 2 = further history. *: Significant difference versus cases 4 and 5 (p < .01), **: Significant difference versus case 4 (p < .05). ^#^: Significant difference male versus female (p < .05), °: Significant difference part 1 versus part 2 (p < .01).

Female participants were rated to be more empathetic across all cases (Table 5) with a significant difference versus male participants in cases 1 and 3 (p < .001). When data from all female standardized patients (cases 1, 3, and 4.1) were combined, female participants were rated to be significantly more empathetic (p < .01) than male participants (mean 14.2%, 95% CI 12.3-16.3 versus mean 19.2, 95% CI 16.3-22.6), whereas for combined ratings from male standardized patients (cases 2, 4.2, and 5) no significant difference was found between female and male participants (mean 13.9, 95% CI 12.0-16.1 versus mean 15.0, 95% CI 12.7-17.7).

**Table 5:** Empathy evaluation by CARE questionnaire

	**Female participants**		**Male participants**	
	**Mean (%)**	**95% CI**	**Mean (%)**	**95% CI**
**Total**	13.7*	11.9 – 15.7	16.6	14.3 – 19.2
**Case 1**	14.9*	11.1 – 20.1	23.2	16.9 – 31.7
**Case 2**	15.9	11.9 – 21.4	18.5	13.5 – 25.3
**Case 3**	12.0*	9.2 – 15.6	17.5	13.2 – 23.3
**Case 4.1**	12.6	9.4 – 16.9	14.0	10.2 – 19.2
**Case 4.2**	11.6	8.7 – 15.6	11.6	8.5 – 15.9
**Case 5**	15.7	11.7 – 21.1	17.0	12.4 – 23.3

*: Significant difference versus male participants (p < .001).

**Figure 1:**
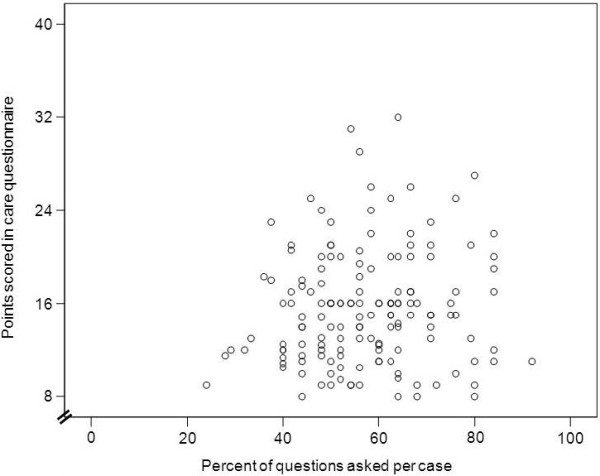
**Correlation between CARE questionnaire points and percentage of history questions per case and participant.** No correlation between empathy scores and percentage of questions asked by participants can be detected (r = 0.093, p = .26, n = 150).

Furthermore, the mixed models regression analysis as well as the Pearson correlation analysis revealed no correlation (r = 0.093, p = .26) between the percentages of medical aspects asked per case and participants’ respective empathy score in the CARE questionnaire (Figure 1). Also, no correlation could be found for overall CARE scores of each participant individually with the number of medical details asked in history part 1 or 2 when calculated separately (data not shown). There was also no correlation between empathy in the individual student encounter and the information volunteered by the standardized patient (data not shown).

## Discussion

In our study, medical students near graduation taking histories from five standardized patients only asked for 56.4% of aspects experts regarded as relevant for clinical reasoning. This seems very low and resembles findings from 1976 [23]. It could also be a sign of a lack of clinical reasoning skills in our participating students. Considering the fact that on average 76% of medical diagnoses can be made simply by taking a patient’s history [4], improvement of our students’ clinical reasoning skills to support prompting of questions lead by hypotheses seems badly needed. Bordage showed that clinical reasoning skills and the way knowledge is organized in memory play an important role for successful history taking [24]. The art of combining medical knowledge and a changing list of possible differential diagnoses as history taking progresses can be taught and learned [24]. That students need to learn to ask “the right” questions to come up with differential diagnoses has been demonstrated in another study where diagnostic accuracy was higher when case vignettes contained all required patient information versus case vignettes in the chief complaint format where questions have to be asked to obtain further information to make a diagnosis [25].

In daily practice a patient will not know which information is important for the physician to exercise clinical reasoning. Hence, questions in history taking are usually developed from a chief complaint. However, clinical reasoning skills are not systematically taught in our six-year medical curriculum [19]. Interestingly, in case 3 (58-year old woman with abdominal pain due to perforated sigma diverticulitis) participants asked for 78.6% of her symptoms (part 1) which are taught in two thematic blocks while the other four patients’ diseases are only taught in one thematic block, respectively. As repetition has been demonstrated to reinforce key concepts [26], repetitive training with patients who present with abdominal pain in two thematic blocks could have had an effect on gathering important medical information during history taking for this particular patient in our study.

Communication skills, measured by the CARE questionnaire were overall quite good in our study with a mean of 13.7 points for female and 16.6 points for male participants (total range of points: 8 = best score, 40 = worst score). This can be considered a satisfying result regarding observations that physicians’ communication style is associated with improved patient satisfaction, increased patient adherence with medications and improved medical outcomes [27]. Another noteworthy result was that female standardized patients rated female participants’ empathy significantly higher compared with male participants’ empathy. Virtual medical visits have shown that female patients were more satisfied with female physicians’ more caring communication style [28]. Interestingly, in another study female students were also rated significantly higher than male students at OSCE stations with female standardized patients [29]. As discussed by the authors, this might also reflect more patient-centred interactions and communication patterns of female students [30] or different perception of empathy by female patients like we observed in our study. It might be helpful to raise the awareness in medical students during communication training that differences in male and female communication can occur. Identifying these differences could further improve empathetic communication of male and female students.

In contrast to our original assumption that empathetic participants might gather a higher number of relevant medical information we found no correlation between the amount of important medical information gathered with empathy scores given by standardized patients. Even though these two features resemble different constructs this result is somewhat surprising as we thought that the concept of empathy might be supportive for information gathering. However, whilst we have not shown that students who are more empathic gain more information, we have also shown that they do not gain any less information. It still seems somewhat disconcerting that a physician might be rated “very empathetic” by a patient but has not been educated enough in clinical reasoning to gather enough information to make the correct diagnosis. However, since effective physician-patient communication has been observed to improve patient health outcomes [31] the concurrence of high quality information gathering and empathy seems to be of great importance and should be taken into account in curricular planning. As asking relevant questions and empathetic communication are not simply two sides of the same coin but need both to concur in successful patient encounters we propose the following: gathering information using clinical reasoning skills and empathetic communication may be acquired in separate courses. Students, however, also need training opportunities and feedback for the combined approach. Observation of the student-patient interaction and the clinical reasoning based hypothetical-deductive approach taken by the student to lead to differential diagnoses are often the last opportunity to receive feedback on history taking and diagnostic skills [32]. This could also be combined with feedback from standardized patients and other observers on empathy and other communication skills needed for interaction with patients [33] to prevent the rise and fall of students’ skills obtaining a medical history while they pass through the medical curriculum [6].

One limitation of our study is that data were only analysed at one medical school. The mediocre amount of medical information gathered from history taking and the non-correlation with empathy scores rated by standardized patients might not be seen under other circumstances. For instance, students from a vertically integrated curriculum have been shown to feel better prepared for their postgraduate training [34]. Furthermore, selecting participants based on a “first come, first served” basis might have preselected for very motivated students and the total number of participants is small. Also, standardized patients’ training in rating on the CARE questionnaire was only validated for one medical school. Additionally, the amount of symptoms voluntarily presented by standardized patients was significantly different which might have lead to a certain bias in the scores. Furthermore, even though the items for the checklists were generated by an expert panel it needs to be addressed that research has shown that experts, when it comes to their own practice do not actually complete all the items themselves that they defined as important [35]. Therefore, our checklists could have included too many items leading to lower total results for the students.

## Conclusions

Asking relevant questions to gather important medical information using clinical reasoning and empathy are two sides of the coin of history taking. Both skills need to be acquired during medical school training. Their eventual combination resembles the daily routine of practising physicians and seems to be the key for successful and effective medical care provided to patients. Therefore, training situations for medical students are desirable where they have the opportunity to be observed exercising both skills and receiving feedback for further improvement.

## Abbreviations

CI: Confidence interval; OSCE: Objective structured clinical examination.

## Competing interests

The authors have no conflicts of interest to declare.

## Authors’ contributions

All authors have contributed sufficiently to the project to be included as authors: MWM and SH designed the study, FO and SH piloted the instrument for the video ratings, FO and DV acquired the data, FO and SS performed the statistical analysis. FO and SH drafted the manuscript. All authors read and approved the final manuscript.

## Pre-publication history

The pre-publication history for this paper can be accessed here:

http://www.biomedcentral.com/1472-6920/13/67/prepub

## References

[B1] Kassirer J Wong J Kopelman R Learning clinical reasoning 2010 2 Baltimore: Lippincott Williams & Wilkins

[B2] Lown B The lost art of healing: practicing compassion in medicine 1999 New York: Ballantine Books

[B3] Hampton JR Harrison MJG Mitchell JRA Richard JS Seymour C Relative contributions of history-taking, physical examination, and laboratory investigation to diagnosis and management of medical outpatients BMJ 1975 2 486 489 10.1136/bmj.2.5969.486 1148666 PMC1673456

[B4] Peterson MC Holbrook JH Von Hales D Smith NL Staker LV Contributions of the history, physical examination, and laboratory investigation in making medical diagnoses West J Med 1992 156 163 165 1536065 PMC1003190

[B5] Alois JF Jonas E Skills in history-taking and physical examination J Med Educ 1976 51 410 415 1263231 10.1097/00001888-197605000-00009

[B6] Pfeiffer C Madray H Ardolino A Willms J The rise and fall of students’ skill in obtaining medical history Med Educ 1998 32 283 288 10.1046/j.1365-2923.1998.00222.x 9743783

[B7] Kurtz S Silverman J Benson J Draper J Marrying content and process in clinical method teaching: enhancing the Calgary-Cambridge guides Acad Med 2003 78 802 809 10.1097/00001888-200308000-00011 12915371

[B8] Schlechter GP Blank LL Godwin HA LaCombe MA Novack DH Rosse WF Refocusing on history-taking skills during internal medicine training Am J Med 1996 101 210 216 10.1016/S0002-9343(96)80078-7 8757362

[B9] Stewart M Brown JB Donner A McWhinney IR Oates J Weston WW Jordan J The impact of patient-centered care on outcomes J Fam Pract 2000 49 796 804 11032203

[B10] Buller MK Buller DB Physicians’ communication style and patient satisfaction J Health Soc Behav 1987 28 375 388 10.2307/2136791 3429807

[B11] Vågan A Medical students’ perceptions of identity in communication skills training: a qualitative study Med Educ 2009 43 254 259 10.1111/j.1365-2923.2008.03278.x 19250352

[B12] Helfer RE An objective comparison of the pediatric interviewing skills of freshman and senior medical students Pediatrics 1970 45 623 627 5438165

[B13] Klamen DL Williams RG The effect of medical education on students‘ patient-satisfaction ratings Acad Med 1997 72 57 61 10.1097/00001888-199710001-00020 9008571

[B14] Elwyn G Arriving at the postmodern medical consultation Eur J Gen Pract 2004 10 93 97 10.3109/13814780409044542 15534573

[B15] Coulter A Elwyn G What do patients want from high-quality general practice and how do we involve them in improvement Br J Gen Pract 2002 52 S22 S26 12389766 PMC1316137

[B16] Salmon P Mendick N Young B Integrative qualitative communication analysis of consultation and patient and practitioner perspectives: towards a theory of authentic caring in clinical relationships Patient Educ Couns 2011 82 448 454 10.1016/j.pec.2010.10.017 21111558

[B17] Ramani S Promoting the art of history taking Med Teach 2004 26 374 376 10.1080/01421590410001683230 15203853

[B18] Van Thiel J Kraan HF Van der Vleuten CPM Reliability and feasibility of measuring medical interviewing skills: the revised Maastricht history-taking and advice checklist Med Educ 1991 25 224 229 10.1111/j.1365-2923.1991.tb00055.x 1857278

[B19] Van den Bussche H Anders S Ehrhardt M Göttsche T Hüneke B Kohlschütter A Kothe R Kuhnigk O Neuber K Rijntjes M Quellmann C Harendza S Is a reformation oft he medical training worthwhile? The quality of the Hamburg curriculum under the old and the new board certification law Z Arztl Fortbild Qualitatssich 2005 99 419 423 [Article in German] 16277056

[B20] Wijnen-Meijer M Van der Schaaf M Booij E Harendza S Boscardin C Van Wijngaarden J Ten Cate TJ An argument-based approach to the validation of UHTRUST: can we measure how recent graduates can be trusted with unfamiliar tasks? Adv Health Sci Educa Theory Pract 2013 Feb 12 [Epub ahead of print] 10.1007/s10459-013-9444-x 23400369

[B21] Neumann M Wirtz M Bollschweiler E Warm M Wolf J Pfaff H Psychometric evaluation of the German version of the "Consultation and Relational Empathy" (CARE) measure at the example of cancer patients Psychother Psychosom Med Psychol 2008 58 5 15 [Article in German] 10.1055/s-2007-970791 17429761

[B22] Nendaz MR Gut AM Perrier A Reuille O Louis-Simonet M Junod AF Vu NV Degree of concurrency among experts in data collection and diagnostic hypothesis generation during clinical encounters Med Educ 2004 38 25 31 10.1111/j.1365-2923.2004.01738.x 14962023

[B23] Maguire GP Rutter DR History-taking for medical students. I-Deficiencies in performance Lancet 1976 2 558 560 60632 10.1016/s0140-6736(76)91804-3

[B24] Bordage G Elaborated knowledge: a key to successful diagnostic thinking Acad Med 1994 69 883 885 10.1097/00001888-199411000-00004 7945684

[B25] Nendaz MR Raetzo MA Junod AF Vu NV Teaching Diagnostic Skills: Clinical Vignettes or Chief Complaints? Adv Health Sci Educ Theory Pract 2000 5 3 10 10.1023/A:1009887330078 12386472

[B26] Paige JT Kozmenko V Yang T Gururaja RP Hilton CW Cohn I Jr Chauvin SW Attitudinal changes resulting from repetitive training of operating room personnel using of high-fidelity simulation at the point of care Am Surg 2009 75 584 590 19655602

[B27] Stewart M Meredith L Brown JB Galajda J The influence of older patient-physician communication on health and health-related outcomes Clin Geriatr Med 2000 16 25 36 10.1016/S0749-0690(05)70005-7 10723615

[B28] Schmid Mast M Hall JA Roter DL Disentangling physicians sex and physician communication style: their effects on patient satisfaction in a virtual medical visit Patient Educ Couns 2007 68 16 22 10.1016/j.pec.2007.03.020 17482418

[B29] Carson JA Peets A Grant V McLaughlin K The effect of gender interactions on students’ physical examination ratings in objective structured clinical examination stations Acad Med 2010 85 1772 1776 10.1097/ACM.0b013e3181f52ef8 20881825

[B30] Krupat E Hiam C Fleming MZ Freeman P Patient-centeredness and its correlates among first year medical students Int J Psychiatry Med 1999 29 347 356 10.2190/DVCQ-4LC8-NT7H-KE0L 10642908

[B31] Stewart MA Effective physician-patient communication and health outcomes: a review Can Med Assoc J 1995 152 1423 1433 7728691 PMC1337906

[B32] Dare AJ Cardinal A Kolbe J Bagg W What can the history tell us? An argument for observed history-taking in the trainee intern long case assessment N Z Med J 2008 121 51 57 18815604

[B33] Troncon LEA Structured, three-way, role-play activity for improving history-taking skills Med Educ 2009 43 1081 1117 10.1111/j.1365-2923.2009.03521.x 19788643

[B34] Wijnen-Meijer M Ten Cate OTJ Van der Schaaf M Borleffs JCC Vertical integration in medical school: effect on the transition to postgraduate training Med Educ 2010 44 272 279 10.1111/j.1365-2923.2009.03571.x 20444058

[B35] Rethans JJ Norcini JJ Barón-Maldonado M Blackmore D Jolly BC LaDuca T Lew S Page GG Southgate LH The relationship between competence and performance: implications for assessing practice performance Med Educ 2002 36 901 909 10.1046/j.1365-2923.2002.01316.x 12390456

